# Use-dependent potentiation of voltage-gated calcium channels rescues neurotransmission in nerve terminals intoxicated by botulinum neurotoxin serotype A

**DOI:** 10.1038/s41598-017-16064-3

**Published:** 2017-11-20

**Authors:** Phillip H. Beske, Katie M. Hoffman, James B. Machamer, Margaret R. Eisen, Patrick M. McNutt

**Affiliations:** Department of Neuroscience, U.S. Army Medical Research Institute of Chemical Defense, Gunpowder, MD 21010 USA

## Abstract

Botulinum neurotoxins (BoNTs) are highly potent toxins that cleave neuronal SNARE proteins required for neurotransmission, causing flaccid paralysis and death by asphyxiation. Currently, there are no clinical treatments to delay or reverse BoNT-induced blockade of neuromuscular transmission. While aminopyridines have demonstrated varying efficacy in transiently reducing paralysis following BoNT poisoning, the precise mechanisms by which aminopyridines symptomatically treat botulism are not understood. Here we found that activity-dependent potentiation of presynaptic voltage-gated calcium channels (VGCCs) underlies 3,4-diaminopyridine (3,4-DAP)-mediated rescue of neurotransmission in central nervous system synapses and mouse diaphragm neuromuscular junctions fully intoxicated by BoNT serotype A. Combinatorial treatments with 3,4-DAP and VGCC agonists proved synergistic in restoring suprathreshold endplate potentials in mouse diaphragms fully intoxicated by BoNT/A. In contrast, synapses fully intoxicated by BoNT serotypes D or E were refractory to synaptic rescue by any treatment. We interpret these data to propose that increasing the duration or extent of VGCC activation prolongs the opportunity for low-efficiency fusion by fusogenic complexes incorporating BoNT/A-cleaved SNAP-25. The identification of VGCC agonists that rescue neurotransmission in BoNT/A-intoxicated synapses provides compelling evidence for potential therapeutic utility in some cases of human botulism.

## Introduction

Botulinum neurotoxins (BoNTs) are a closely related family of protein neurotoxins expressed by members of the *Clostridium* genus of anaerobic bacteria^1^. Collectively, the BoNTs are the most poisonous substances known, with estimated human LD_50_ values as low as 0.1–1 ng/kg. The neurotoxins are categorized by antigenic properties into seven serotypes (BoNT/A-G), with highly conserved structural and functional properties^2^. All BoNT serotypes are initially expressed as 150 kDa proteins, which are activated by proteolytic cleavage into 100 kDa heavy chains (HC) and 50 kDa light chains (LC) that remain associated through a disulfide bond. The C-terminal of HC mediates highly selective and efficient binding to endosomal receptors on the presynaptic membrane of peripheral neurons. Following internalization via synaptic endocytosis, the N-terminal domain of HC forms a pore that facilitates translocation of LC through the endosomal membrane to the pre-synaptic cytosol^3^. The translocated LC refolds to form a zinc-dependent endoprotease that specifically targets and cleaves fusogenic SNARE proteins essential for neurotransmitter exocytosis. SNAP-25 is targeted and cleaved by BoNT/A, /C, and /E; synaptobrevin1/2 (SYB) is cleaved by BoNT/B, /D, /F, and /G; and syntaxin-1 (STX1) is cleaved by BoNT/C reviewed in^2^. BoNT proteolysis of neuronal SNARE proteins interferes with formation of the Ca^2+^-activated fusogenic complex required for fast neurotransmission, thereby blocking synaptic vesicle fusion. Symptoms of BoNT poisoning clinically manifest as a descending flaccid paralysis that results in death by asphyxiation once respiratory muscles are compromised. The only treatment options for clinical botulism are supportive care, such as mechanical ventilation and parenteral feeding, until neuromuscular function is restored. Some serotypes produce paralysis that persists for months, requiring sustained supportive care with increased risk of co-morbidities.

Although post-exposure prophylaxis with antitoxin can efficiently neutralize toxin prior to neuronal uptake^4,5^, antitoxin administration requires clinical evidence of progressive paralysis, and thus even patients that receive a timely administration of antitoxin are likely to suffer symptoms of botulism. Despite intensive research efforts over the past three decades, small molecule LC inhibitors with clinically suitable pharmacokinetics and pharmacodynamics have not been identified^6,7^. Consequently, there remains a critical need for treatments that reverse neuromuscular paralysis in botulism patients.

*In vitro* studies suggest that neuromuscular function of intoxicated tissues can be transiently improved by enhancing neuronal Ca^2+^ influx, such as through increased extracellular Ca^2+^, treatment with aminopyridines or exposure to cationic ionophores^8–10^. Of these, the aminopyridines are particularly intriguing because derivatives such as 4-aminopyridine and 3,4-diaminopyridine (3,4-DAP) are currently in clinical use for neuromuscular indications^11^. Aminopyridines block the intracellular domain of voltage-gated K^+^ channels (VGKC), extending the duration of action potential-induced presynaptic depolarization and increasing neurotransmitter release^12,13^. However, clinical evaluations of aminopyridines in patients with severe serotype A and B botulism have resulted in highly variable and conflicting reports of efficacy^14–16^. Because the precise mechanisms by which aminopyridines enhance muscle contraction in paralyzed tissues are not known, conditions under which they can mitigate botulism symptoms have not been established.

While augmenting presynaptic cytosolic Ca^2+^ influx is likely to increase release probability at non-intoxicated release sites in partially intoxicated NMJs^17^, it is unknown whether other mechanisms can also contribute to symptomatic rescue. Here, we investigated the cellular and molecular mechanisms of 3,4-DAP-mediated rescue of neurotransmission in networked cultures of primary rat neurons and isolated mouse diaphragms intoxicated by BoNT serotypes A, D or E (the serotypes most commonly associated with human disease). These studies were conducted under conditions in which neurotransmission was thoroughly blocked by BoNT intoxication, thereby enabling the evaluation of treatments that restore the ability of intoxicated synapses to undergo synaptic release. These data provide compelling evidence that enhancing presynaptic Ca^2+^ influx through agonism of voltage-gated Ca^2+^ channels (VGCCs) is sufficient to rescue neurotransmission in synapses intoxicated by BoNT/A. Furthermore, they reveal that drugs that synergize with 3,4-DAP to enhance phasic Ca^2+^ influx represent a potentially novel class of candidate botulism therapeutics with improved efficacy profiles.

## Results

### 3,4-DAP rescue of evoked neurotransmission is unique to BoNT/A-poisoned synapses and dependent on VGCC activation

**Figure 1:**
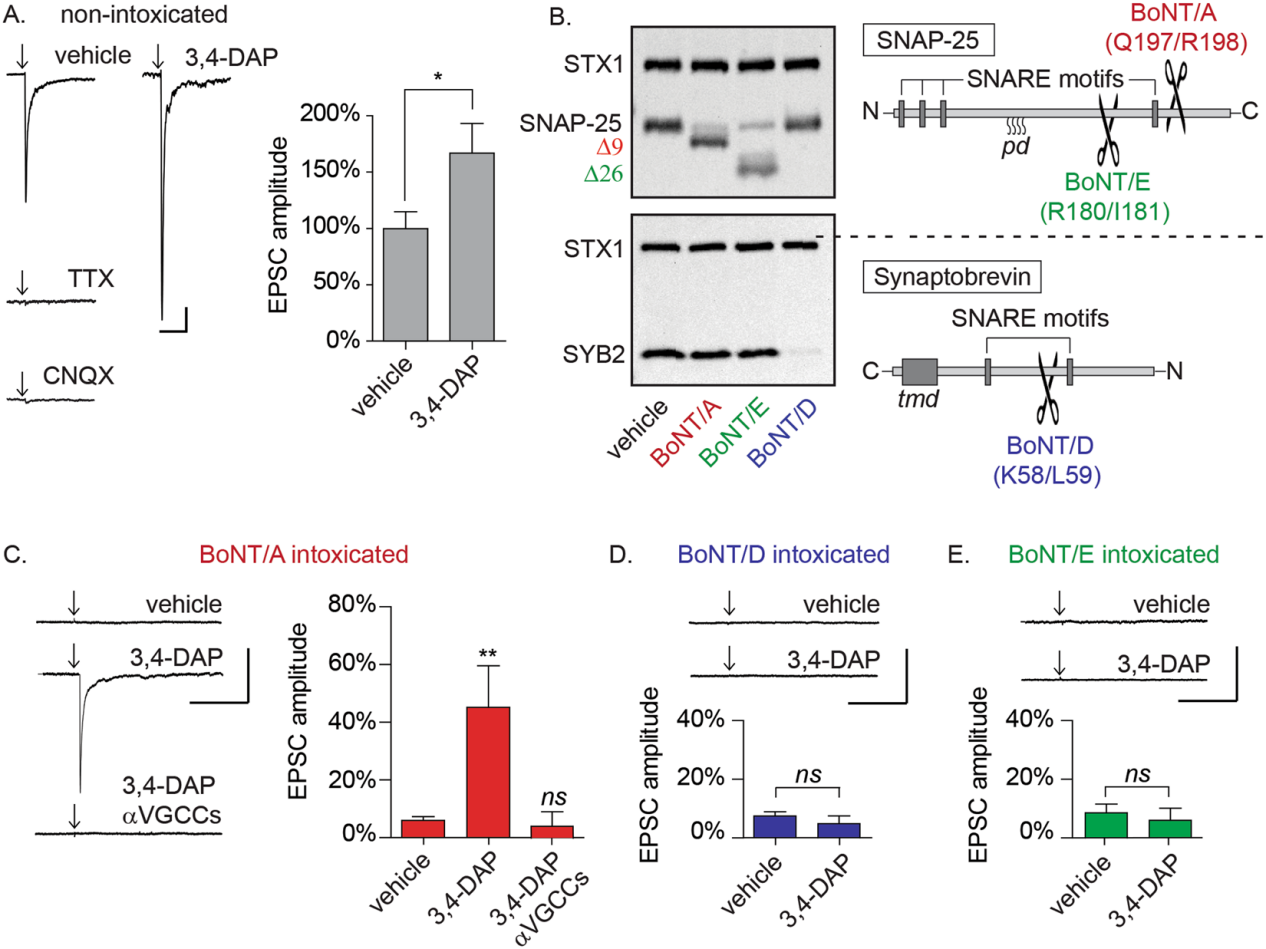
3,4-DAP restores evoked synaptic release in neuronal cultures intoxicated by BoNT/A but not BoNT/D or BoNT/E. (**A**) Representative whole-cell recordings of evoked neurotransmission in the presence of vehicle, TTX (5 µM), CNQX (20 µM), or 3,4-DAP (50 µM) and quantitation of normalized EPSC amplitudes versus vehicle (n = 6 each for TTX and CNQX; n = 16 for 3,4-DAP). (**B**) Representative Western blots of SNAP-25, STX1 and SYB2 from lysates harvested from primary neuron cultures treated with vehicle, BoNT/A, BoNT/D, or BoNT/E treatment and schematic of SNAP-25 and SYB structure with BoNT/A, /D and /E cleavage sites. Each panel representes a complete image from blots stained for STX1 and SNAP-25 (top) or SYB2 (bottom). Note that while proteolytic cleavage fragments of SNAP-25 can be immunologically detected, cleavage of SYB2 results in the rapid degradation of the N-terminal fragment, and therefore loss of reactivity is considered evidence of intoxication^19^. (**C**–**E**): Representative whole-cell recordings and of evoked neurotransmission and mean EPSC amplitudes in cultures intoxicated by BoNT/A, BoNT/D, or BoNT/E and treated with vehicle (n = 12), 3,4-DAP (50 µM; n = 12 for BoNT/A and n = 9 for BoNT/D or E) or 3,4-DAP plus VGCC antagonists (n = 8). Mean EPSC amplitudes are normalized to recordings from age-matched, non-intoxicated control cultures. Arrows represent stimulation times. Scale bars represent 400 ms (x-axis) and 100 pA (y-axis). All data presented as mean ± SEM. *Indicates *p* < 0.05, **Indicates *p* < 0.01, *ns* = not significant.

To characterize the effects of 3,4-DAP on action potential-elicited neurotransmission at intoxicated synapses, whole-cell patch-clamp electrophysiology was used to measure excitatory postsynaptic currents (EPSCs) in response to extracellular stimulation of proximal axons in primary neuron cultures^18^. These studies were done in the presence of GABA and NMDA receptor antagonists, permitting aggregate analysis of synaptic transmission at afferent glutamategic synapses. Field stimulation of primary rat hippocampal and cortical neurons at 18–24 d after plating produced EPSCs with amplitudes of 512.2 ± 77.1 pA (Fig. 1A). Spontaneous miniature EPSCs (mEPSCs) measured in the same neuron population had mean amplitudes of 14.8 ± 1.1 pA, indicating that field stimulation elicited release of approximately 35 quanta per trial. The elimination of EPSCs by addition of either tetrodotoxin (TTX) or the AMPA/kainic acid receptor antagonist CNQX confirmed that EPSCs constituted post-synaptic responses to presynaptic release (Fig. 1A). 3,4-DAP treatment of naïve cultures increased EPSC amplitudes by 166.7 ± 26.4% (*p* = 0.027; Fig. 1A), without altering spontaneous miniature EPSCs (mEPSC) amplitudes or frequencies (Supplementary Figure S1A), consistent with the known pharmacological role of 3,4-DAP as a voltage-gated potassium channel blocker.

The effects of 3,4-DAP on neurotransmission were first tested in cultured neurons intoxicated with high concentrations of BoNT/A^19^. This approach allowed us to specifically test for treatments that restored synaptic function at fully intoxicated synapses. Western blot analysis of whole cell lysates revealed conversion of SNAP-25 to the BoNT/A cleavage product SNAP-25Δ9 in intoxicated cultures (Fig. 1B). BoNT/A intoxication reduced EPSC amplitudes to 4.0 ± 1.3% of naïve controls, demonstrating near-complete blockade of evoked release (Fig. 1C). 3,4-DAP treatment restored evoked release in 100% of evaluated neurons, increasing ESPC amplitudes to 45.1 ± 14.4% of naïve controls (*p* = 0.0012; Fig. 1C). As expected, 3,4-DAP had no effect on intoxicated neurons in the presence of TTX, indicating that synaptic rescue by 3,4-DAP was not mediated post-synaptically (Supplementary Figure S1B).

We next evaluated the molecular mechanisms involved in 3,4-DAP rescue of neurotransmission. A secondary neuropharmacological effect of 3,4-DAP is prolongation of voltage-gated Ca^2+^ channel (VGCC) activation, which increases presynaptic Ca^2+^ influx and vesicle release probability^12^. This suggested that enhanced Ca^2+^ influx through VGCCs in response to 3,4-DAP treatment contributed to the rescue effect. To test this hypothesis, BoNT/A-intoxicated cultures were treated with 3,4-DAP and L, N, R and P/Q-type VGCC antagonists (nimodipine, ω-agatoxin IVA and ω-conotoxin MVIIC). The presence of VGCC antagonists completely blocked rescue of neurotransmission by 3,4-DAP, demonstrating a requirement for VGCC activity (*p* = 0.029; Fig. 1C).

We next evaluated whether 3,4-DAP rescued EPSCs in cultures intoxicated by BoNT serotypes D and E. Unlike BoNT/A, which cleaves nine residues from the C-terminus of SNAP-25, BoNT/E removes an additional 17 amino acids, functionally eliminating the C-terminal SNARE domain. BoNT/D cleaves 56 residues from the N-terminal of rat synaptobrevin-2 (SYBΔ56), also eliminating the C-terminal SNARE domain (Fig. 1B)^10^. Intoxication with BoNT/D or BoNT/E resulted in proteolytic cleavage of SNAP-25 and SYB, respectively (Fig. 1B), and depressed EPSCs similar to BoNT/A (Fig. 1D,E). However, unlike BoNT/A-intoxicated synapses, 3,4-DAP had no effect on EPSCs in neurons intoxicated with serotype D (Fig. 1D; 7.5 ± 1.5% versus 5.0 ± 2.7%; *p* = 0.40) or E (Fig. 1E; 8.6 ± 3.0% versus 6.0 ± 4.1%, *p* = 0.61).

### VGCC agonism is sufficient to rescue neurotransmission in synapses intoxicated by BoNT/A

The above data demonstrated that VGCC activation was necessary for 3,4-DAP rescue of neurotransmission in BoNT/A-intoxicated synapses. To more specifically determine whether VGCC currents were necessary for rescue, we recorded spontaneous synaptic release (mEPSCs) in the presence of elevated Ca^2+^ as well as VGCC agonists. In contrast to evoked release, spontaneous release is a probabilistic phenomenon, and consequently, mEPSC frequencies reflect the average release probability among the hundreds or thousands of afferent synapses on each neuron^20,21^. The ability to surveil the behavior of many synapses, with single-synapse resolution, facilitated a more specific evaluation of the effects of treatment conditions on release probabilities^22^.

**Figure 2:**
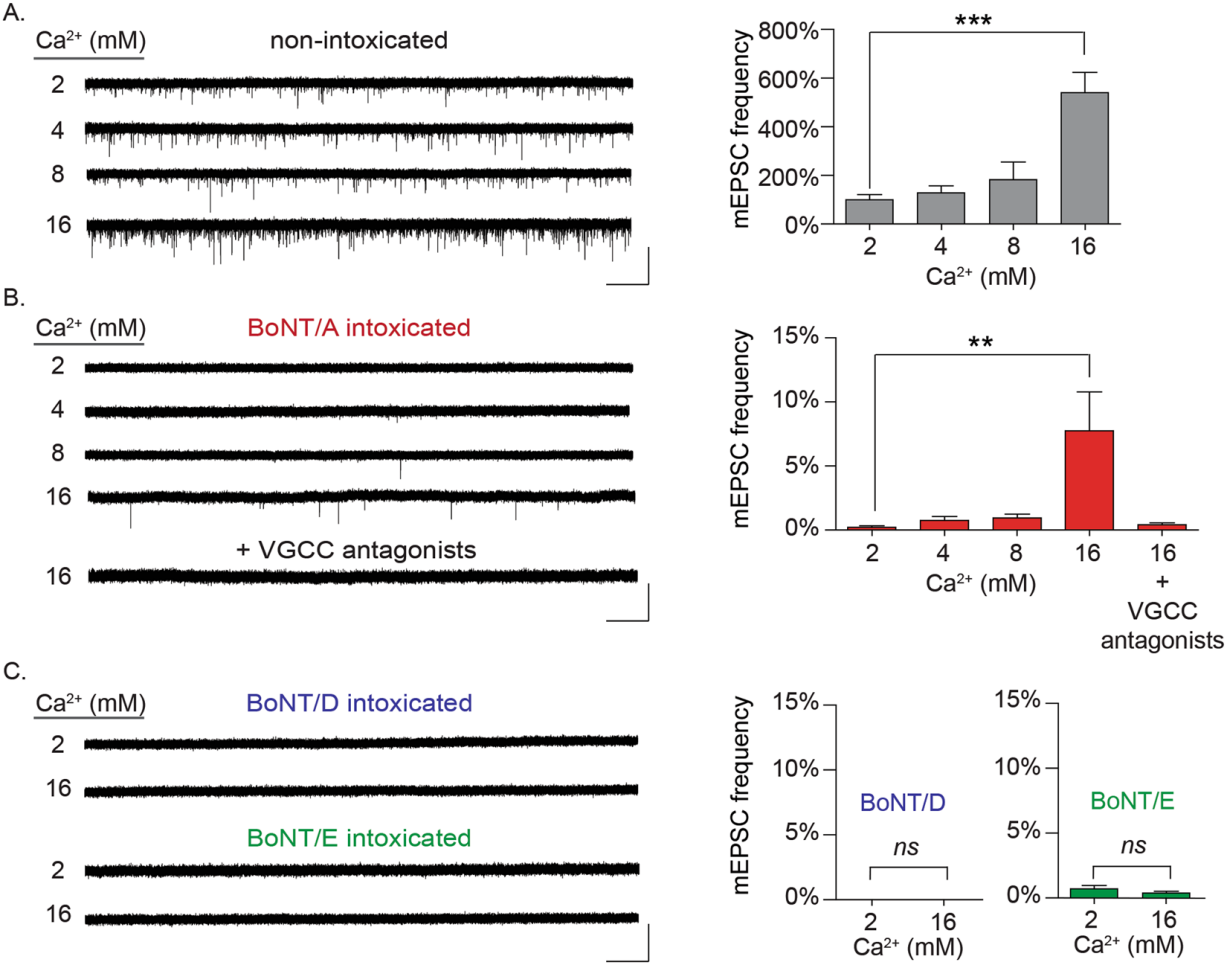
Rescue of spontaneous synaptic activity in BoNT/A-silenced synapses by elevated extracellular Ca^2+^ requires VGCCs. (**A**) Representative whole-cell recordings and mean mEPSC frequencies from non-intoxicated cultures in the presence of 2, 4, 8, or 16 mM Ca^2+^. Representative whole-cell recordings and mean mEPSC frequencies from BoNT/A-intoxicated cultures, in the presence of 2, 4, 8, or 16 mM Ca^2+^ as well in 16 mM Ca^2+^ with VGCC antagonists (10 µM nimodipine, 0.5 µM ω-agatoxin IVA and 0.5 µM ω-conotoxin MVIIC). (**C**) Representative whole-cell recordings and mean mEPSC frequencies from neurons intoxicated by BoNT/D or BoNT/E and incubated in 2 or 16 mM Ca^2+^. mEPSC frequencies are normalized to recordings from age-matched, non-intoxicated control cultures. Scale bars represent 5 s (x-axis) and 40 pA (y-axis). All data presented as mean ± SEM and n ≥ 10 neurons for all conditions. **Indicates *p* < 0.01, ***Indicates *p* < 0.001.

To evaluate whether enhanced Ca^2+^ influx was sufficient to rescue spontaneous release from intoxicated synapses, mEPSCs were first recorded from intoxicated neurons in the presence of 2–16 mM extracellular Ca^2+^ (Fig. 2A,B). Consistent with previous studies, intoxication with BoNT/A reduced mEPSC frequencies by 99.98 ± 0.01% versus non-intoxicated controls in physiological Ca^2+^ (*p* < 0.001)^19^. Although 4 or 8 mM extracellular Ca^2+^ had no effect on mEPSC rates (*p* = 0.93 and 0.46, respectively), addition of 16 mM Ca^2+^ increased mEPSC frequencies to 7.8 ± 0.9% of controls (*p* < 0.01; Fig. 2B), without affecting mEPSC amplitudes as compared to naïve controls (16.4 ± 1.3 versus 15.4 ± 0.9 pA, *p* = 0.52). Addition of VGCC antagonists eliminated the recovery of mEPSCs in 16 mM Ca^2+^ (Fig. 2B; 0.4 ± 0.2%, *p* < 0.0001), confirming that the rescue effect required Ca^2+^ influx through VGCCs. In contrast, 16 mM Ca^2+^ did not restore spontaneous release in neuron cultures intoxicated with BoNT/D or BoNT/E (Fig. 2C).

**Figure 3:**
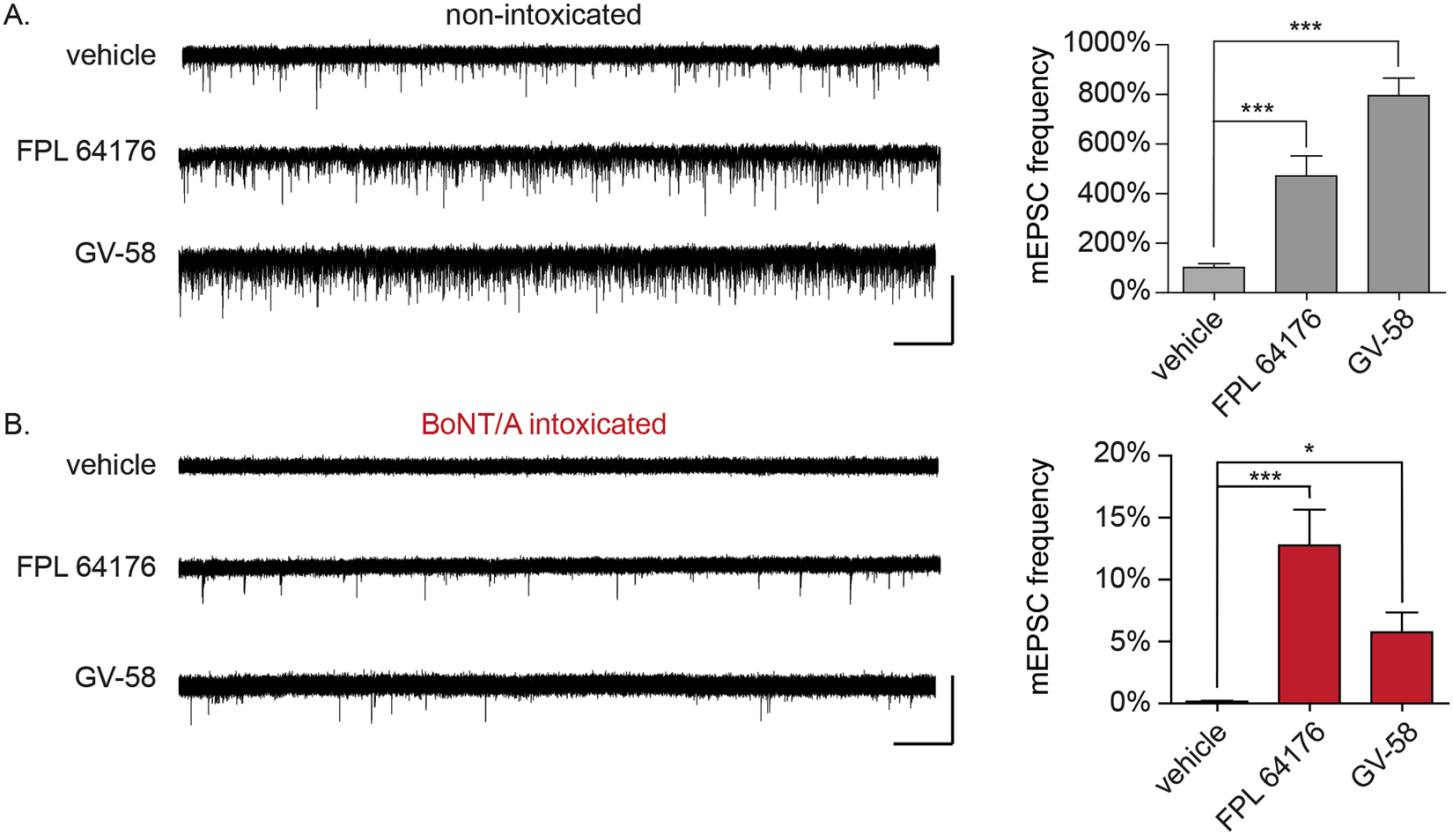
Use-dependent L-type or N/P/Q-type VGCC agonists restore spontaneous synaptic activity to neuronal cultures fully intoxicated by BoNT/A in physiological Ca^2+^. (**A**) Representative whole-cell recordings and mean mEPSC frequencies in non-intoxicated cultures treated with vehicle, FPL 64176 (10 µM) or GV-58 (50 µM). (**B**) Representative whole-cell recordings and mean mEPSC frequencies in BoNT/A-intoxicated cultures treated with vehicle, FPL 64176 (10 µM) or GV-58 (50 µM). mEPSC frequencies are normalized to recordings from age-matched, non-intoxicated control cultures. Scale bars represent 5 s (x-axis) and 40 pA (y-axis). All data presented as mean ± SEM and n ≥ 12 neurons for all conditions. *Indicates *p* < 0.05, ***Indicates *p* < 0.001.

**Figure 4:**
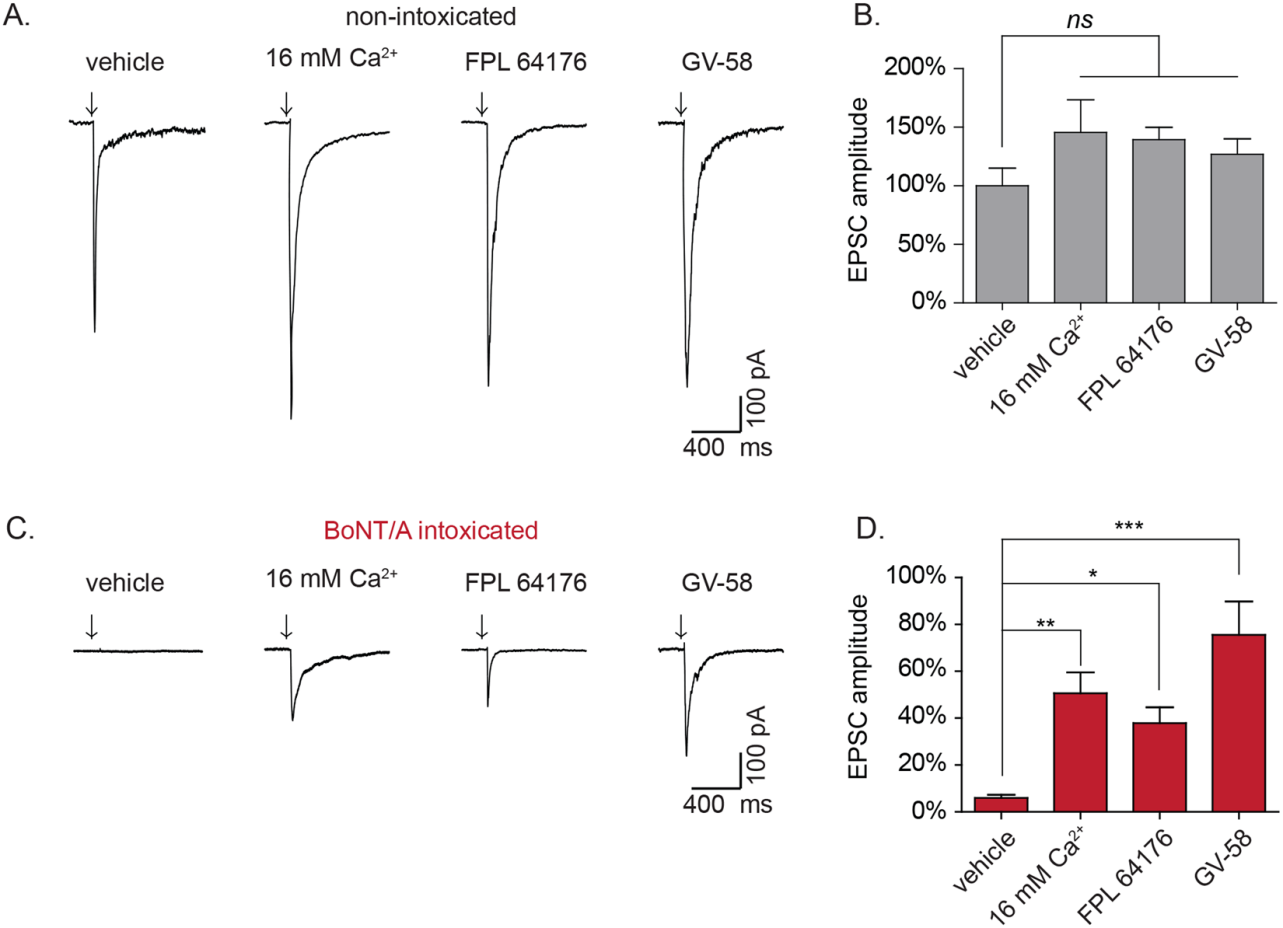
Enhanced presynaptic Ca^2+^ influx rescues evoked release in BoNT/A-intoxicated cultures. (**A**) Representative whole-cell recordings of evoked neurotransmission in non-intoxicated cultures treated with vehicle, 16 mM Ca^2+^, FPL 64176 (10 µM), or GV-58 (50 µM). (**B**) Mean EPSC amplitudes from non-intoxicated cultures. (**B**) Representative whole-cell recordings of evoked neurotransmission in BoNT/A-intoxicated neurons treated with vehicle, 16 mM Ca^2+^, FPL 64176 (10 µM), or GV-58 (50 µM). (**D**) Mean EPSC amplitudes for BoNT/A-intoxicated cultures. EPSC amplitudes are presented as mean ± SEM and n ≥ 12 recordings for all conditions. Scale bars represent 400 ms (x-axis) and 100 pA (y-axis). Arrows represent stimulation times. *Indicated *p* < 0.05, **Indicates *p* < 0.01, ***Indicates *p* < 0.001, *ns* = not significant.

While blockade of VGCCs proved to prevent rescue under multiple contexts, it remained possible that this was a reflection of the critical role that Ca^2+^ plays in neurotransmission, and not directly related to the rescue phenomenon. Thus, we next sought to establish whether agonists of VGCCs could rescue neurotransmission in the presence of physiological Ca^2+^. For these studies, the selective VGCC agonists GV-58 (N-, P/Q-types) or FPL 64176 (FPL; L-type) were added to BoNT/A-intoxicated neurons and spontaneous and evoked release were quantified. In non-intoxicated neurons, GV-58 and FPL increased mEPSC production by 469.4 ± 80.7% and 783.0 ± 60.4%, respectively (Fig. 3A) without affecting mEPSC amplitudes (Supplementary Figure S1B), confirming their presynaptic effects. In BoNT/A-intoxicated neurons, FPL increased mEPSC production from 0.15 ± 0.01% to 12.7 ± 2.9% (*p* < 0.0001), while GV-58 increased mEPSC production to 5.7 ± 1.6% (*p* = 0.03, Fig. 3B). Similarly, FPL increased EPSC amplitudes in BoNT/A-intoxicated neurons by 50.6 ± 8.9% (*p* < 0.05 versus vehicle-treated controls) while GV-58 increased EPSC amplitudes by 75.6 ± 14.3% (*p* < 0.001 versus vehicle-treated controls; Fig. 4A–D).

### Combination treatment with 3,4-DAP and GV-58 restores EPSC amplitudes in neurons intoxicated by BoNT/A, but not BoNT/D or /E

**Figure 5:**
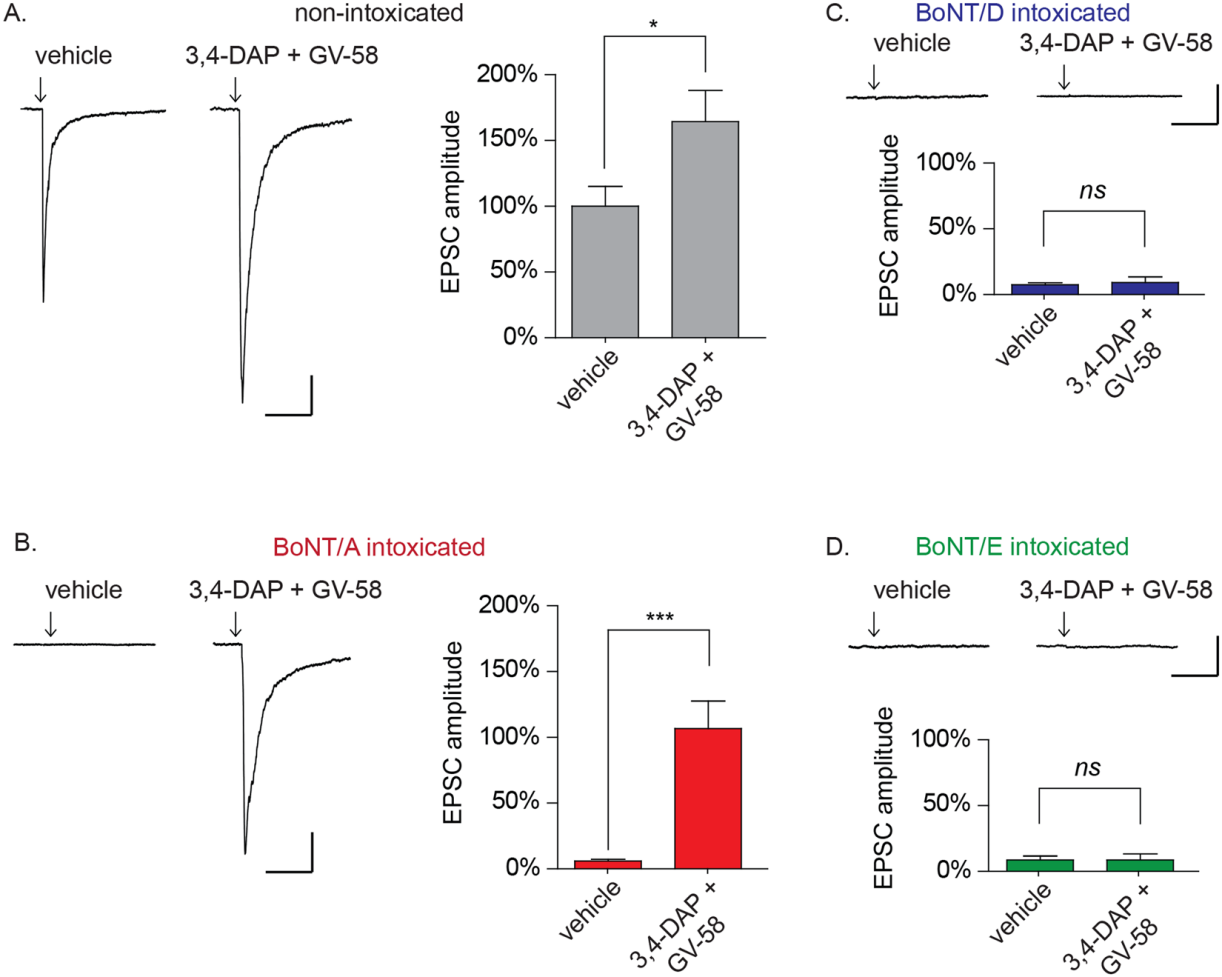
3,4-DAP plus GV-58 restores evoked neurotransmission to baseline levels in cultured neurons intoxicated by BoNT/A, but not BoNT/D or BoNT/E. Representative EPSC and mean EPSC amplitudes from cultures intoxicated with: (**A**) vehicle; (**B**) BoNT/A; (**C**) BoNT/D; or (**D**) BoNT/E. Arrows represent stimulation times. Scale bars represent 400 ms (x-axis) and 100 pA (y-axis). Mean EPSC amplitudes are presented as mean ± SEM; n ≥ 13 recordings for 5A-B and n ≥ 9 recordings for 5C–D. *Indicates *p* < 0.05, ***Indicates *p* < 0.001, *ns* = not significant.

We next evaluated whether combinatorial addition of 3,4-DAP and a VGCC agonist had additive effects on evoked release in BoNT/A-intoxicated cultures. GV-58 was chosen for these studies because it specifically targets VGCCs that are expressed at the synapse and associated with evoked release^23,24^. In BoNT/A-intoxicated neurons, combination treatment with 3,4-DAP and GV-58 restored EPSC amplitudes to levels that were indistinguishable from non-intoxicated cultures (Fig. 5A,B; 106.6 ± 21.0% versus 100 ± 15.1% respectively, *p* = 0.80), and 17-fold improved over intoxicated cultures treated with vehicle alone (6.0 ± 1.3%, *p* < 0.001). Combination treatment proved more effective than 3,4-DAP alone (*p* = 0.019), though there was no statistical improvement compared to GV-58 alone (*p* = 0.41).

Combination treatment with 3,4-DAP and GV-58 had no effect on evoked release in BoNT/D-intoxicated cultures (*p* = 0.67; Fig. 5C) or BoNT/E-intoxicated cultures (*p* = 0.99; Fig. 5D), further illustrating that VGCC-mediated rescue of neurotransmission is specific to BoNT/A-intoxicated synapses.

### 3,4-DAP plus GV-58 restores suprathreshold release in mouse diaphragm endplates fully intoxicated by BoNT/A

Although these data demonstrated that enhanced VGCC activity could restore neurotransmission to CNS synapses severely intoxicated by BoNT/A, the physiological target of botulism is peripheral neurons. Thus, we used intracellular endplate recordings to evaluate the effects of treatment with 3,4-DAP and/or GV-58 on phrenic nerve-elicited endplate potentials (EPPs) in mouse hemidiaphragms preparations intoxicated *ex vivo* by BoNT/A or BoNT/E. The hemidiaphragm assay closely mimics the physiological progression of respiratory paralysis, and has been used extensively to study toxin mechanisms of action^25,26^.

**Figure 6:**
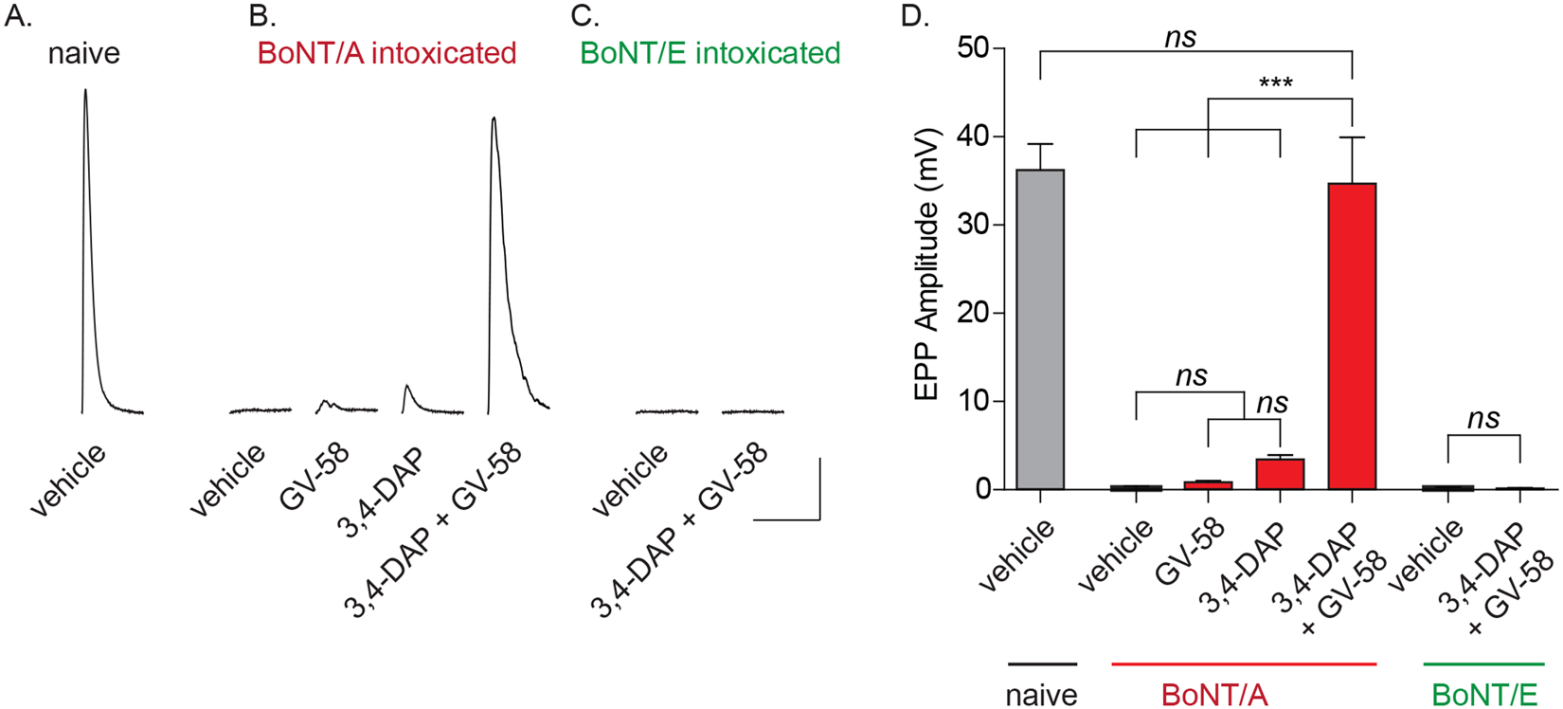
3,4-DAP plus GV-58 has superadditive effects in restoring robust EPP production in mouse diaphragm endplates fully intoxicated by BoNT/A. Representative evoked endplate recordings (top) and corresponding mean EPP amplitudes (in mV, bottom) from non-intoxicated and BoNT/A-intoxicated mouse phrenic nerve-hemidiaphragm preparations treated with vehicle, GV-58 (50 µM), 3,4-DAP (2 µM), or GV-58 plus 3,4-DAP (50 µM and 2 µM, respectively); and BoNT/E-intoxicated tissue treated with vehicle, or GV-58 plus 3,4-DAP. Representative traces are the average of six EPPs elicited from single endplates. Scale bars represent 25 ms (x-axis) and 5 mV (y-axis). Grouped data is presented as mean ± SEM (n = 4–6 diaphragm per condition; 5 endplates recorded per diaphragm). ***Indicates *p* < 0.001, *ns* = not significant.

To reproduce the comprehensive synaptic blockade used in CNS cultures, diaphragms were intoxicated with high doses of BoNT/A or /E and EPPs were serially monitored in sequential endplates until phrenic nerve stimulation failed to elicit EPPs in five consecutive endplates. This represented the statistical point where the majority of endplates are fully intoxicated, and few or no release sites remained competent to undergo vesicle fusion. Addition of either 3,4-DAP (2 µM) or GV-58 (50 µM; doses established in pilot dose-ranging studies) to BoNT/A-intoxicated preparations rapidly restored sub-threshold release, producing EPPs that were 3.49 ± 0.44 mV and 0.89 ± 0.11 mV, respectively (*p* < 0.01; Fig. 6). Surprisingly, combinatorial addition of 3,4-DAP plus GV-58 had a supra-additive effect in BoNT/A-intoxicated endplates, producing EPPs with amplitudes of 34.7 ± 5.19 mV (Fig. 6). In contrast, 3,4-DAP plus GV-58 had no apparent effect on diaphragms intoxicated by BoNT/E (Fig. 6), consistent with previous data suggesting that VGCC-mediated rescue was specific to BoNT/A-intoxicated synapses.

## Discussion

In this study, we investigated the cellular and molecular mechanisms responsible for 3,4-DAP-mediated rescue of neurotransmission with the goal of determining the conditions under which 3,4-DAP could be effective in restoring synaptic function. We found that 3,4-DAP rescue of synaptic release in intoxicated synapses was specifically mediated through VGCC activity. Furthermore, treatments that either increased Ca^2+^ influx through VGCCs or prolonged VGCC opening times were sufficient to rescue evoked and spontaneous release in BoNT/A-intoxicated neuron cultures. Functional rescue of neurotransmission was unique to BoNT/A and did not occur with serotypes D or E under any circumstance. The physiological relevance of these findings were confirmed in diaphragm NMJs, where combination treatment with 3,4-DAP and GV-58 proved synergistic, restoring putatively suprathreshold release to hemidiaphragms comprehensively paralyzed by BoNT/A.

The finding that rescue was unique to BoNT/A-intoxicated synapses is consistent with biochemical data suggesting that SNAP-25Δ9 remains competent to engage in or facilitate vesicle fusion^27^. In *in vitro* vesicle fusion studies, SNAP-25Δ9 directly engages with SYB1/2 and STX1 to mediate low-efficiency vesicle fusion. In contrast, vesicle fusion is abolished by substitution with the BoNT/E-cleavage product SNAP-25Δ26, illustrating that SNAP-25Δ9 and SNAP-25Δ26 have functionally distinct abilities to engage in neurotransmission. Deletion studies conducted in BoNT/A-intoxicated PC12 adrenal chromaffin cells found that overexpression of SNAP-25Δ9 restores low-level catecholamine release^28^. Collectively, these data suggest that SNAP-25Δ9 can directly participate in vesicle fusion, albeit with reduced efficiency compared to intact SNAP-25.

Although low-efficiency engagement of SNAP-25Δ9 in vesicle release provides a plausible mechanistic basis for rescue of neurotransmission in BoNT/A-intoxicated cultures, we cannot exclude the possibility that a compensatory release mechanism is activated in response to VGCC agonism. However, this putative mechanism must still rely on SNAP-25Δ9, otherwise rescue would have been observed in synapses intoxicated with BoNT/D or /E. Examples of such a mechanism could include direct stabilization of SNAP-25Δ9 in the fusogenic complex by coordination with Ca^2+^ ions or recruitment of an accessory protein. However, synthetic vesicles incorporating SYB, STX1 and SNAP-25Δ9 are capable of supporting vesicle fusion in the absence of Ca^2+^ or external protein factors, suggesting that neither Ca^2+^ nor Ca^2+^-regulated accessory proteins are required for fusion activity^29^. Alternatively, we propose that increased Ca^2+^ flux through activated VGCCs in the presence of 3,4-DAP, VGCC agonists or elevated extracellular Ca^2+^ promotes rescue of neurotransmission by prolonging the kinetics of release. Synaptic release is initiated by local increases in Ca^2+^, which cause the Ca^2+^ sensor synaptotagmin (SYT) to undergo a conformation change that permits assembly of SYB, STX1 and SNAP-25 into the fusogenic *trans*-SNARE configuration^30,31^. In the absence of Ca^2+^, SYT is a negative regulator of vesicle fusion, thus the duration of SYT permissiveness is determined by the magnitude and duration of local Ca^2+^ transients^17^. This leads us to hypothesize that 3,4-DAP and/or VGCC agonists effectively extend the temporal window during which SNARE-mediated fusion is molecularly permissible by increasing presynaptic Ca^2+^ transients. This would prolong the window during which less efficient fusion events can occur, such as those involving SNAP-25Δ9.

The ability of use-dependent VGCC agonists to rescue neurotransmission has therapeutic implications for stages of botulism other than complete paralysis. For example, most CNS neurons have 1–3 release sites per synaptic bouton^32^, whereas typical motor nerve terminals contain 800–1200 distinct release sites^33^. Continuous single-endplate recordings reveal the progressive depression of evoked release in response to BoNT intoxication^34^, illustrating that partially intoxicated endplates contain a mixture of intact and intoxicated release sites. Since 3,4-DAP treatment increases EPSC amplitudes in both non-intoxicated and BoNT/A-intoxicated synapses, we hypothesize that enhanced Ca^2+^ influx will increase neurotransmission from partially intoxicated motor nerve terminals through two distinct mechanisms: increased release probability at non-intoxicated release sites, which would occur in a serotype-independent fashion; and rescue of fusion at release sites associated with SNAP-25Δ9. If correct, Ca^2+^-mediated rescue of neurotransmission will then be influenced by factors such as the serotype involved and the extent of paralysis. These factors may contribute to the highly variable and conflicting results reported in the small number of clinical studies using aminopyridines as post-symptomatic treatments for serotype A and B botulism^14–16,35^.

Adult NMJs and CNS synapses are believed to predominantly rely on N-type (Ca_v_2.2) and P/Q-type (Ca_v_2.1) VGCCs for synaptic transmission^23,24^. These two VGCC subtypes incorporate synaptic protein interaction domains that interact with Stx1 and SNAP-25, placing the channels in close proximity to docked synaptic vesicles, and providing a structural association between voltage-gated Ca^2+^ influx and synaptic vesicle fusion^36,37^. Although N- and P/Q-type VGCCs are often co-located within the presynaptic compartment, they appear to have functional distinctions. For example, in detonator synapses N-type VGCCs contribute to multivesicular release at specific release sites, whereas P/Q-type VGCCs drive widespread synaptic release^38^. In contrast, neuronal L-type VGCCs (Ca_v_1.2 through Ca_v_1.4) have been proposed to gate Ca^2+^-release from internal Ca^2+^ stores, activating signaling processes that indirectly modulate presynaptic function^39,40^. L-type VGCCs are primarily believed to mediate somatic Ca^2+^ transients^41,42^, although several studies suggest that they also directly contribute to neurotransmission^43,44^. While our data indicate that L- or N/P/Q-type VGCC agonists can promote release from naïve as well as BoNT/A-intoxicated CNS synapses, we did not determine whether release is exclusively mediated by direct modulation of Ca^2+^ transients at release sites, or whether indirect Ca^2+^ signaling mechanisms are also involved. Moreover, since central synapses, motor endplates and autonomic synapses are likely to exhibit morphological and functional differences in VGCC activity, VGCC agonists may have synapse type-specific effects on rescue of neurotransmission.

Although aminopyridines have been studied as BoNT countermeasures for decades, their clinical potential has been compromised by adverse CNS effects at presumptive therapeutic doses^45,46^. Therefore, there is interest in reducing the central access of 3,4-DAP, or alternatively in identifying co-treatments that allow reduction in 3,4-DAP concentrations to tolerable levels while retaining or improving therapeutic efficacy^47,48^. While the combination of VGCC agonists and 3,4-DAP appeared very robust in rescuring synaptic release, direct modulation of Ca^2+^ influx carries the risk of neurotoxic responses. For example, excessive cytosolic Ca^2+^ levels can activate compensatory changes in presynaptic function that reduce Ca^2+^ sensitivity^49^ or cause presynaptic Ca^2+^ toxicity^50^. However, most VGCC agonists are use-dependent, enhancing Ca^2+^ influx predominantly during action potential firing^51,52^. This results in the temporally constrained influx of Ca^2+^ at specific subcellular compartments, as opposed to tonic Ca^2+^ currents. For similar reasons, combinatorial use of 3,4-DAP and GV-58 is under preclinical evaluation as a treatment for myasthenic diseases^53^. However, it should be noted that although both FPL and GV-58 produced encouraging *in vitro* results as BoNT/A treatments, *in vivo* use of both drugs is limited by their poor aqueous solubility and there is a need for derivatives with improved pharmacokinetic and/or pharmacodynamics profiles.

In conclusion, we demonstrate that enhanced Ca^2+^ influx through VGCCs restores neurotransmission to BoNT/A-intoxicated synapses. Our findings suggest that co-administration of use-dependent VGCC agonists with 3,4-DAP may increase the safety and efficacy of treatments to prolong survival during the acute phase of botulism, or alternatively, to accelerate recovery of muscle function during the chronic phase of botulism.

## Experimental procedures

### Animal use statement

The experimental protocol was approved by the Animal Care and Use Committee at the United States Army Medical Research Institute of Chemical Defense (USDA certificate number 51-F-0006). All procedures were conducted in accordance with the principles stated in the Guide for the Care and Use of Laboratory Animals and the Animal Welfare Act of 1966 (P.L. 89–544), as amended.

### Reagents/Supplies

BoNT/A1 (2.5 × 10^8^ MLD_50_/mg), BoNT/D1 (2.8 × 10^7^ MLD_50_/mg) and BoNT/E1 (3.0 × 10^7^ MLD_50_/mg) were purchased from Metabiologics (Madison, WI, USA). For BoNT/E, toxin was activated by a 60 min incubation at 37 °C with 0.3 mg/mL TPCK-treated trypsin in 0.05 M sodium phosphate buffer (pH 6.5)^19^. ω-agatoxin IVA, ω-conotoxin MVIIC and GV-58 were purchased from Alomone Labs (Jerusalem, Israel). FPL 64176 was purchased from Tocris Bioscience (Bristol, UK). All reagents for electrophysiology buffers were obtained from Sigma-Aldrich (St. Louis, MO, USA).

### Neuronal culture and BoNT intoxication

Fresh E18 rat hippocampal, cortical and ventricular zone tissues were obtained from BrainBits (Springfield, IL, USA), dissociated according to the manufacturer’s instructions, and plated at a density of 125,000 cells/cm^2^ on polyethylenimine/laminin-coated glass coverslips (Sigma-Aldrich). Neuronal cultures were maintained at 5% CO_2_, 37 °C, and 95% humidity in NbActiv4 medium (BrainBits). Experiments were performed 16 to 24 days after plating. For neuronal intoxication, BoNT/A, BoNT/D or BoNT/E were prepared at 100x final concentration in fresh NbActiv4 medium and added to neuron cultures at 100 pM. Neurons were analyzed 20–24 hours after addition of BoNT.

### Whole-cell patch clamp electrophysiology

Experiments were performed to record spontaneous AMPAR-mediated miniature excitatory post-synaptic currents (mEPSCs) as previously described^19^. Briefly, neurons were bathed in extracellular recording buffer containing (in mM): 140 NaCl, 3.5 KCl, 1.25 NaH_2_PO_4_, 2 CaCl_2_, 1 MgCl_2_, 10 Glucose, 10 HEPES, 5 TTX, 20 bicuculline, and 10 MK-801 (pH = 7.3; 315 ± 10 mOsm/Kg). Patch pipettes (5–10 MΩ) were fabricated from borosilicate glass (Sutter Instruments, Novato, CA) and filled with an intracellular recording buffer containing (in mM): 140 K-gluconate, 5 NaCl, 2 Mg-ATP, 0.5 Li-GTP, 0.1 CaCl_2_, 1 MgCl_2_, 1 EGTA and 10 HEPES (pH = 7.3; 315 ± 10 mOsm/Kg). Whole-cell −70 mV voltage-clamp recordings were collected at 20–22 °C with an EPC10 (Heka, Lambrecht/Pfalz, Germany) and Heka Patchmaster 2.53 software. mEPSC frequency were detected from whole-cell recordings using Mini-Analysis v6 (Synaptosoft Inc., Fort Lee, NJ).

For evoked AMPAR-medicated excitatory post-synaptic currents (eEPSCs), neurons were bathed in extracellular recording buffer without TTX. Recording electrodes were filled with an intracellular recording buffer containing (in mM): 140 CsCl, 5 NaCl, 2 Mg-ATP, 0.5 Li-GTP, 0.1 CaCl_2_, 1 MgCl_2_, 1 EGTA, 10 HEPES, and 10 QX-314 (pH 7.3; 315 ± 10 mOsm/Kg). After establishing whole-cell configuration, the intracellular solution was allowed to equilibrate for 2 min. A concentric bipolar stimulating electrode (FHC, Bowdoin, ME) was placed 100–150 µm from the neuron to be recorded and a train of 10 stimulations (1.0 ms, 0.9 mA, 0.1 Hz) was applied to stimulate presynaptic fibers. The mean amplitude of the first three EPSCs was calculated using Axograph X software (Axograph.com).

### Endplate electrophysiology

For *ex vivo* diaphragm studies, Male C57BL/6 mice (6–10 wk; Jackson Labs, Bar Harbor, ME) were group-housed and provided a standard diet with regular enrichment and water *ad libitum*. Mice were thoroughly anesthetized using 5% isoflurane and euthanized by decapitation. Diaphragm muscles and corresponding phrenic nerves were isolated from euthanized mice by dissection at 22–24 °C in Tyrode’s solution (137 mM NaCl, 5 mM KCl, 1.8 mM CaCl_2_, 1 mM MgSO_4_, 24 mM NaHCO_3_, 1 mM NaH_2_PO_4_ and 11 mM D-glucose, pH 7.4). After dissection, muscles were attached with dissection pins to a 10-mm tissue culture dish containing Sylgard (Dow Corning; Midland, MI, USA) and the preparation was perfused with oxygenated Tyrode’s. Muscle viability was verified using a 0.2 ms squarewave of direct current at suprathreshold amplitudes to the phrenic nerve using a bipolar stimulating electrode driven by a stimulation isolation unit (Digitimer North America; Ft. Lauderdale, FL, USA).

Muscle potentials were recorded on a HEKA Elektronik EPC10 patch clamp amplifier using sharp glass electrodes (10–20 MΩs) pulled with a Sutter Instrument P1000. Muscles fibers were impaled close to endplate junctions and recordings with a starting resting membrane potential (RMP) > −60 mV were rejected. Muscle contraction was selectively blocked with 1 μM μ-conotoxin GIIIB, which preferentially blocks muscle-specific voltage-activated Na^+^ channels (Alamone Labs). For all experimental conditions, recordings were performed at 22–24 C in perfused oxygenated Tyrode’s. EPPs were elicited by suprathreshold phrenic nerve stimulation with a square wave of constant current for 0.2 ms at 0.05 Hz. EPP amplitudes were averaged from 6 consecutive stimulations per muscle fiber and corrected for non-linear summation using the following conversion: EPP /1-(0.8 * EPP /(V_m_ - V_rev_))^54^. For this correction we used experimentally determined values of V_m_ (the resting membrane potential) and 0 mV for the V_rev_ (reversal potential). For each hemidiaphragm, corrected EPP amplitudes from 5 endplates were averaged to obtain a single value.

Naïve recordings were performed after 30 minutes of equilibration post-dissection. Hemidiaphragms were intoxicated by bath addition of 670 pM of BoNT/A, or 210 pM BoNT/E coupled with suprathreshold phrenic nerve stimulation at 0.5 Hz for 30 min, followed by persistent 0.05 Hz stimulation. These concentrations and conditions were sufficient to produce rapid and comprehensive synapse failure within 3–4 hours. Comprehensive intoxication was determined to be the point where consistent stimulation failure was observed in six stimulations (0.05 Hz) per endplate, observed across 5 consecutive endplates. To characterize the effects of 3,4-DAP and GV-58 on endplate function, the hemidiaphragm preparations were incubated with 2 µM of 3,4-DAP and/or 50 µM GV-58 in oxygenated Tyrode’s for 45 minutes prior to recordings.

For all electrophysiology experiments, current measurements were filtered online at 2.9 kHz and digitized at 10 kHz. Data analysis and graphing were performed in Prism v6.1 (GraphPad software, La Jolla, CA).

### Western blot analysis

Protein from neuronal cultures was harvested, quantified by BCA (Pierce), separated by SDS-PAGE and transferred to PVDF. Blots were stained and imaged as previously described^19^. Primary antibodies used for Western blotting included SNAP-25 (Covance, Princeton, NJ), STX1, and SYB2 (Synaptic Systems, Gottingen, Germany). Blots were fluorescently probed using a goat anti-mouse secondary conjugated to Alexa Fluor 488 (Invitrogen, Carlsbad, CA) for detection of STX1 (33 kDa), SYB2 (12 kDa) and SNAP-25 (18, 24 or 25 kDa).

### Statistical analyses

For each patched neuron, evoked amplitudes were averaged among the first three consecutive stimulations to produce the mean evoked current per neuron (in pA). Mean evoked amplitude values were then averaged among neurons within each condition and presented as percentages of age- and lot-matched control neurons. mEPSC frequencies were calculated by determining the mean frequency (in Hz) of events measured during 180 s of continuous voltage-clamp recording. Spontaneous release rates measured in BoNT-intoxicated neurons were normalized to mean frequencies observed in age- and lot-matched, vehicle-treated neurons and presented as percentages of control values. Statistical significance among means was calculated either using Student’s t-test analysis (for two-sample comparisons), 1-way ANOVA testing (significance from control determined using the Dunnett’s test against the vehicle control population), or 1-way ANOVA testing with Bonferroni’s multiple comparison test. Quantitative data are presented as mean ± the standard error of the mean, with statistical significance listed, or represented using the following indicators: * indicates *p* < 0.05; ** indicates *p* < 0.01; *** indicates *p* < 0.001.

### Data availability

All data generated or analysed during this study are included in this published article (and its Supplementary Information files).

## Electronic supplementary material

Supplementary Figure S1
