# Supplementary figures and images for: Use-dependent potentiation of voltage-gated calcium channels rescues neurotransmission in nerve terminals intoxicated by botulinum neurotoxin serotype A
Source: Sci Rep. 2017 Nov 20;7:15862. doi: 10.1038/s41598-017-16064-3 (PMC5696531; doi:10.1038/s41598-017-16064-3)

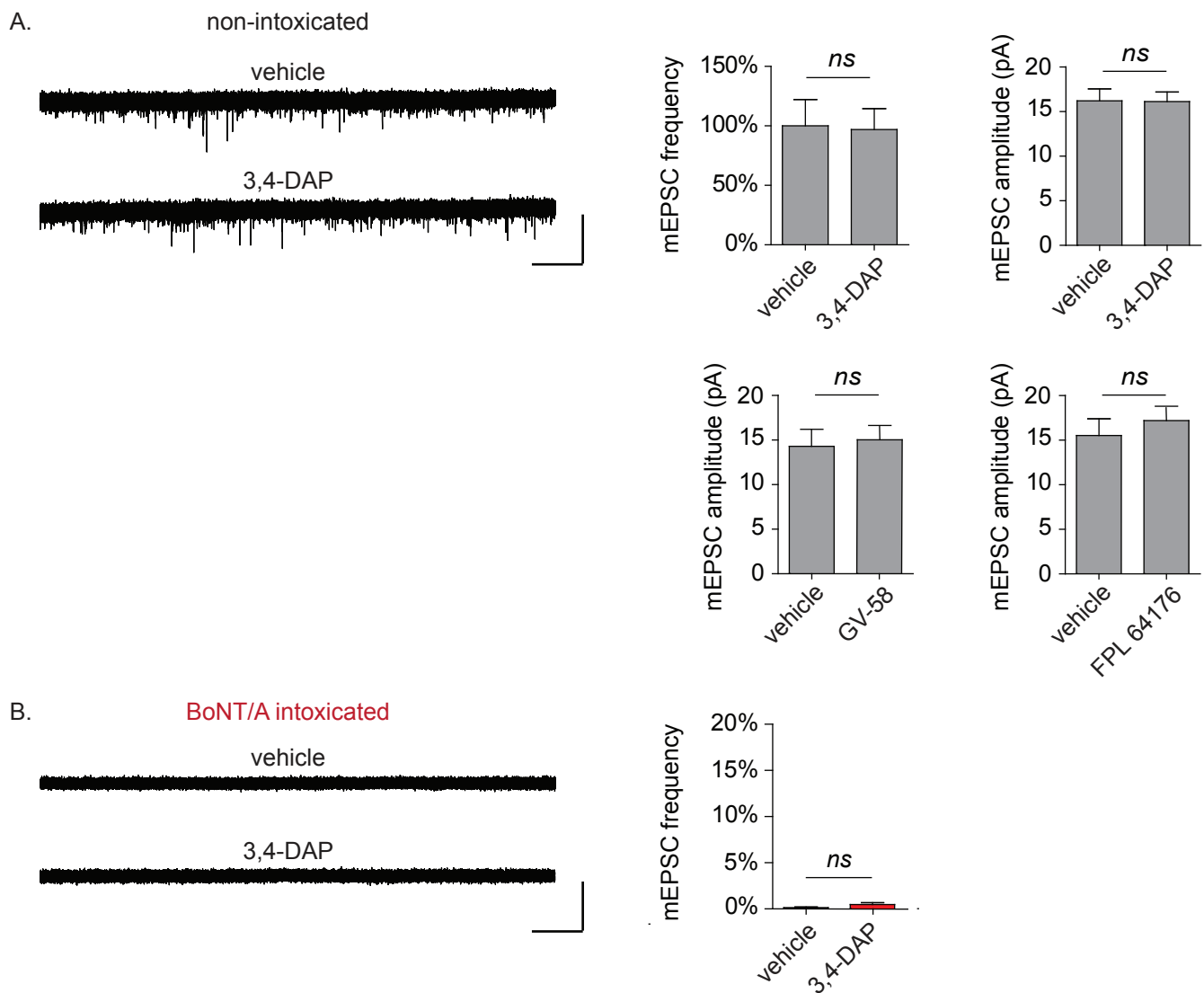

Supplement: Supplementary file 1 — Supplementary Figure S1 [file 41598_2017_16064_MOESM1_ESM.pdf]
